# Barriers to adherence to cytology exam: a case study in low-income Colombian women

**DOI:** 10.1186/s12913-023-09700-4

**Published:** 2023-07-25

**Authors:** Paula C. Bermúdez, Marcela Arrivillaga, Kirvis Torres Poveda, Diana M. Castrillón Libreros, Lorena E. Castillo Castillo, Daniela Neira Acevedo

**Affiliations:** 1 Departamento de Salud Pública y Epidemiología, Pontificia Universidad Javeriana Cali, Cali, Colombia; 2 Oficina de Investigación, Pontificia Universidad Javeriana Cali, Cali, Colombia; 3 Chronic Infections and Cancer Division, National Institute of Public Health, Cuernavaca, Morelos México; 4 Hospital de Siloé Siglo XXI, Red de Salud Ladera Empresa Social del Estado, Cali, Colombia; 5 Instituto de la Mujer de Cuernavaca, México, Cuernavaca, Morelos México

**Keywords:** Cytology, Treatment Adherence and Compliance, early detection of Cancer, Cervical neoplasms, Secondary Prevention

## Abstract

**Background:**

Cervical cytology is essential for the early detection of cervical cancer. However, in Colombia, only 50% of women with subsidized health insurance were screened in 2019, compared to 100% of women with contributory insurance. This disparity highlights significant barriers that must be addressed. This study aimed to identify the factors that contribute to or hinder adherence to cervical cytology screening among low-income women with subsidized health insurance in a public primary care network in Cali, Colombia, from 2014 to 2018.

**Methods:**

In a qualitative case study, the experience of women and health care and administrative personnel was recovered. Forty-seven women participated in seven focus group discussions. Five other women using the program participated in in-depth interviews. Finally, we interviewed eight people from the healthcare area and the health services administration. The qualitative data collected underwent content analysis, guided by the theoretical framework of Social Determinants of Health. Within this framework, five interconnected dimensions that influence adherence were incorporated.

**Results:**

Adherence is a multifactorial phenomenon, and in relation to attendance at cervical cytology, the analysis delved into the mechanisms that affect it in a low-income context. Barriers to adherence were identified across multiple dimensions, including social and economic factors, health conditions, and patient-related factors, among both adherent and non-adherent women. Among adherent women, barriers and facilitators related to the healthcare team and system, as well as patient-related factors, were identified.

**Conclusions:**

The findings of this research can be useful in developing personalized interventions and strategies to improve adherence and screening outcomes in low-income settings. It is necessary to increase the resources of health insurance entities to establish effective communication channels with women who attend the cervical cancer prevention program.

## Introduction

Cervical cancer poses a significant global public health concern, representing a severe threat to women’s lives [1]. Early detection and treatment through preventive programs are crucial for both survival and quality of life [2, 3]. The projected increase in cervical cancer cases from 570,000 to 700,000 between 2018 and 2030 will result in an annual mortality rise from 311,000 to 400,000. Latin America contributes to over 9% of global cervical cancer cases [4], disproportionately affecting women in low- and middle-income countries, who face limited access to healthcare providers, exacerbating their suffering [5].

Adapting organized cytology-based screening programs, which have proven successful in developed countries, to low-income settings characterized by poverty and barriers to implementing quality national programs, is of utmost importance. These settings have limited capacity and resources to implement cytology-based screening programs or other technologies, and consequently, these settings are associated with higher incidence rates [6]. Effective strategies should evaluate risks and develop educational programs that foster a strong connection between women and prevention efforts [7, 8]. Barriers such as stigma, discrimination, limited knowledge, fear of screening, and strained relationships with healthcare providers hinder access to screening tests [9–11]. Addressing these barriers is essential to enhance the effectiveness of preventive programs and reduce the incidence of cervical cancer.

While adherence studies often focus on biomedical and behavioral aspects, exploring the causes of non-participation, women’s knowledge, beliefs, general and mental health, and coping strategies [12–17], it is crucial to adopt a social perspective that considers broader factors and dimensions to gain a comprehensive understanding [15, 18, 19]. Particularly in low-income settings, a deeper understanding of the social determinants of health (SDH) and adherence mechanisms is essential to generate evidence for designing effective preventive programs [13, 20, 21].

The purpose of the study was to identify the factors affecting the adherence of women to cytology within screening program for cervical cancer detection in low-income settings. Using the theoretical framework of SDH, we will analyze the multidimensionality of adherence, encompassing five dimensions [16]: social/economic, therapy-related, health condition-related, health system/healthcare team, and patient-related factors to contribute to the understanding of SDHs that influence this adherence.

## Methods

### Study design

We conducted a qualitative case study, approved by the Ethics Committees of the Pontificia Universidad Javeriana Cali and the National Institute of Public Health of Mexico. In addition, we have the endorsement of the Ladera Health Network (RSL) as a participant institution. The process to obtain the informed consent of all the participants was in writing once the necessary explanations were made.

### Study context

The city of Cali is in the southwestern part of the country. It has more than 2,300,000 inhabitants. More than 30% are affiliated to the health insurance system subsidized by the state (subsidized regime), which means that they live in conditions of poverty. There are five networks of health service providers that operate the subsidized regime and are public in nature. The RSL serves almost 40% of the poor population and covers 80% of the territory of Cali; its network of hospitals, posts, and health centers covers the entire rural area in 14 townships and six communes in urban areas practically.

### Participants and procedures

We recovered the experience of women beneficiaries of the subsidized regime and assistance and administrative personnel. Forty-seven women participated in seven focus group discussions (FG), and five in-depth interviews (DI); eight other semi-structured interviews (SI) were conducted with RSL staff. For the information approach, we use theoretical sampling. All interviews and FGs were audio-recorded and transcribed into text files. Initially, we did open coding [22]; the data analysis was carried out in parallel to the collection, and the sampling was adjusted to obtain densification of the analysis categories.

Focus groups. At least two FGs were convened for each key topic of interest: (I) adherence to the screening program: according to the Colombian protocol, the women who attended the screening in the last three years are adherents; (II) distance from the home to the place of health care (rural/urban area); (III) and the relationship with primary care services, whether they attended a medical consultation during the last five years. An average of six women participated in each FG.

The invitation to women was carried out in several steps. Initially, we contacted them through women community leaders in the region and through the RSL’s basic primary care teams in rural areas; and we used snowball sampling as a strategy to find new contacts. We invited other women when they leave the medical service centers. Before participation, we confirmed that they met the selection criteria:

- To be affiliated with subsidized regime for the last five years.
- To be between the ages of 25 and 65.
- Have the right to consult the RSL as a reference institution in the first level of complexity during the last five years.
- Have time available to participate in the FG.

FG meetings were held in leaders’ women’s homes and a communal hall close to the RSL facilities. The topics discussed followed the approaches of previously designed guides. At the beginning of each meeting, the research team presented the objectives and purpose of the study, explained what the session would consist of, and proceeded to read the informed consent aloud; subsequently, we obtained the Informed Consent of each participant.

Topics discussed. We investigated the decision to make cytology, family, and partner support in all FGs. In adherent women, we inquired about the care process: Capture mechanisms, procedure (clinical examination, pre, and post), information on cervical cancer prevention, delivery of results, and follow-up (emphasis in cases of alterations). In non-adherent women, we investigated self-care, general state of health, reasons for not attending, and living conditions.

In-depth interviews. We retrieved information from women who did not adhere to the preventive program or health care services or lived in rural areas. The DIs were held in each woman’s home, in a place that allowed privacy for dialogue. The selection criteria, the taking of informed consent, and the topics of the DI were similar to the ones in the FG process, and an interview guide was developed.

Semi-structured interviews. To select the participants from the health services and administration staff, we made a list of 15 people who met the selection criteria. Then, we schedule appointments with each one, considering the time available in which we would be able to apply the previously designed script. The duration of the SI ranged from 30 to 40 min, and they were all recorded in audio and transcribed in text. According to the selection criteria, the interviewees worked with the program for at least four years. They could be professionals or auxiliaries of the RSL essential health teams. Finally, they must have experience in different parts of the territory and have worked in other units such as posts and health centers. The SI explored characteristics of the program, processes of offering and attracting patients, availability of human resources, aspects of the offices, processes and procedures of cancer care, barriers and facilitators for adherence, critical points of the program, and opportunities for improvement.

### Analysis of the information

We used qualitative content analysis based on the women’s speeches recovered during the DI, FG, and some responses from the SI. Information was organized using the Atlas TI software, Version 7.3. It was arranged and classified until finding the meaning in the women’s speeches and the point of view expressed by the officials and health services personnel [23]. The initial nuclei of meaning were established from the emerging categories. Then, fragments were ordered by similarity and grouped into initial codes. Subsequently, groups were reordered obtaining two levels of similar text fragments, according to what the studied aspects of adherence were represented, dividing what is associated with difficulties, reasons or causes and problems for not attending, as a barrier; while everything that motivated or generated the assistance to the screening was categorized as a facilitator.

## Results

A total of 47 women participated in seven FG and five DI. The FG participants had a mean age of 48 years (age range 25 to 63) and all belonged to the poorest strata, with 40% having paid work, mainly in domestic chores. Eight officials from the health services and the administration participated in the DIs, including four professionals and four with technical studies in public health and nursing.

During the analysis several categories emerged related to the participants’ socioeconomic status, living conditions, social support networks. Other categories were related to cultural, religious, and ethnic factors, as well as gender roles. The study also identified complex situations that prevent women from attending cervical cancer prevention programs as an emergent category. Discussing the prevention program allowed the participants to share their experiences, exchange opinions on intimate issues, and discuss the implications of their healthcare decisions. Through anecdotes, the women highlighted their roles as caregivers, while also sharing their experiences using health services. The study’s findings were summarized in Table 1, which includes significant quotes analyzed according to the established categories.

### Life conditions

The study found that a woman’s life conditions play a crucial role in the decisions she makes regarding attending a cervical cancer prevention program. Previous experiences, current conditions, and the trajectory of her life influence her decision-making process. Some SDH can support preventive actions. For example, women acknowledge that having a higher level of education is essential for gaining autonomy, self-empowerment, and taking care of their own health and that of their family. As a result, women may decide to attend the preventive program, postpone it for the future, or choose not to attend.

### Distance to service centers

The distance to healthcare facilities was found to be a significant factor affecting women’s decision to attend cervical cancer screening. Women living in rural and remote areas had varying opinions on where to go for cytology. They preferred to choose the place of care that was most convenient for them. However, the study found that mobile teams visiting their neighborhoods stimulated older women who had never accessed the program before or had low adherence to participate. This was due to the trust they had in the unit that visited their area. It is likely that these women would not have accessed the preventive program if they were invited to urban establishments.

### Religion

The study revealed two distinct perspectives on religion among the women who participated, depending on their beliefs. Some women had a fatalistic perspective and attributed health issues to destiny and faith, rather than with individual behavior or other factors. In contrast, women who regularly accessed health services had a different perspective and saw cytology as a necessary and helpful practice for maintaining their health.

### Ethnicity

During the study, it was observed that indigenous communities in the area have lost many of their traditional practices and beliefs, and their use of health services is similar to that of non-indigenous women. However, it was found that most indigenous women regularly attend screening. While some indigenous women reported problems accessing services, the most significant barrier was identified as their traditional thinking attached to their culture. It was also noted that the Regional Health System did not provide its services with an intercultural approach, which may have contributed to the difficulties experienced by these women.

### Social support

Women attending preventive checkups commonly reported support from their partners. However, some women expressed concern that their partners might have been unfaithful if they were diagnosed with HPV. Other women had a good understanding of the risks associated with HPV infection and the importance of protective measures, even when in a stable relationship.

### Gender role

Women usually accessed the cervical cancer screening through referrals from sexual and reproductive health services. Women seeking intrauterine devices as a method of family planning were referred to cytology screening as part of the protocol. Therefore, the decision to undergo cytology was closely linked to their reproductive and couple roles. Unfortunately, women who were single or approaching the end of their reproductive years were less likely to attend cervical cancer screening.

### Reasons for not attending

The women interviewed reported various reasons for not attending cervical cancer screenings, with family health problems being the primary issue. Many women felt compelled to care for terminally ill, elderly parents, seriously ill children, or both, leading them to put their own health needs aside. Diseases in women also decreased their ability and autonomy to care for themselves. Poverty and weak support networks were also significant reasons for not attending screenings. Additionally, many women expressed fear of pain and embarrassment about being naked in front of someone unknown. Non-adherent women in the focus groups also reported confusion about preventive measures, particularly regarding liquid cytology.

### Perceptions of the cervical cancer prevention program

Based on the interviews conducted, the women reported an overall improvement in the quality of the cervical cancer prevention program in recent years. One aspect they particularly appreciated was the use of only female healthcare providers for obtaining cytology samples. However, the women also highlighted a need for more patient-centered care and greater privacy in clinics as areas for improvement. In addition, the women reported significant variability in the treatment they received from different healthcare providers.

### The administration and health services officials’ point of view

Regarding facilitators: the quality of care in the women’s cancer program was enhanced by the exclusive attention of a doctor. To address the complexity of the relationship between health services and women with abnormal cytology results, the RSL provides support throughout the care process. The staff has made significant efforts to ensure that patient data is up-to-date, and results are delivered promptly.

Regarding the barriers: some women who do not comply with the program may believe that accessing it requires multiple procedures. Others may experience pain during the exams and therefore choose not to return. Forgetfulness has also been observed as a barrier. The standardization of care processes in the RSL facilities is essential to ensure consistency in the quality of care. The slow adoption of new technologies, such as HPV testing, is also problematic, as it is not yet available to the entire population and may be subject to complex procedures imposed by insurance companies. This can create barriers, especially when abnormalities are detected in screening tests. There is also a lack of strategies to attract non-adherent women to preventive programs in general. Finally, internal migration within the territory can pose challenges for follow-up.

**Table 1:** Barriers to adherence to cytology exam: A case study in low-income Colombian women

Category	Quotations	Facilitator/Barrier
Life conditions	“…..I have no life for myself ... my life belongs to my grandchildren and my children… [has a daughter diagnosed with HIV+] I keep an eye on her asking her how she is, and if she has already eaten, and I live with my other daughter, taking care of my grandchildren, the truth is that I don’t have time for myself.” (age 55 years, non adherence)	Barrier
	"I haven’t had time to go for a Pap smear because my mom recently had facial paralysis and I’ve been focused on taking care of her. It’s been stressful for me, and I’ve even had to see a psychiatrist. Sometimes I find myself worrying more about others than myself." (age 45 years, non-adherence)	Barrier
Distance to health services	“…Well, I had [the cytology] done four months ago when a health brigade came to visit us.” (age 55 years, adherence)	Facilitator
	"I haven’t had a Pap smear in 6 years. I used to go to a private clinic that I liked, but now I have to go to a place that’s far away and I don’t like it." (age 61 years, non-adherence)	Barrier
Relationship with health services	“We go to the RSL for checkups, [my husband and my children] every six months… Just like me, to cytology, dentistry, but nothing serious.” (age 40 years, adherence)	Facilitator
Perceptions on the preventive program for cervical cancer	“…Now a woman comes up with something rare in the cytology, and there [follow-up assistants] are looking for them, even under a bridge.” (age 63 years, adherence)	Facilitator
Social support: the role of the partner, the family, and others	“My husband is very aware of my controls, he says to me: well, when is the appointment? Why haven’t you gone?” (30 years, adherence)	Facilitator
Ethnicity	“…My mother also does not go [to the cytology], […], the indigenous people do not show their parts…” (indigenous, age 35 years, non-adherence)	Barrier
	“People say that the indigenous people are careless, I did not study, but I do want my children to be different, they study, I am fighting to give them everything.” (indigenous, age 44 years, adherence)	Facilitator
Religion	“My sister got cancer, and she always attended the cytology, [….] God protects us, no one else.” (age 55 years, non-adherence)	Barrier
Gender role	“…so from the moment you have sex you have to start planning and worrying about your health, your body and everything, right?” (age 44 years, adherence).	Facilitator
Reasons for not attending	“…then one has a flaccid skin like that, and I’m ashamed, I don’t know why one is ashamed to be seen like this without clothes.” (age 38 years, non-adherence).	Barrier
	"I'm terrified of getting a pap smear. It makes me so nervous that I start sweating profusely. I only get it because I know I have to, but it’s a horrible experience. The day before, I can’t even eat because I’m so anxious about it." (age 53 years, non-adherence).	Barrier
	“Five years ago, I had a Pap smear and I haven’t gone back because it made me feel uncomfortable and scared.” (age 55 years, non-adherence).	Barrier

## Discussion

To enhance preventive programs, a comprehensive understanding of the obstacles impeding adherence to cervical cytology is essential. Our research findings offer valuable insights for designing interventions that effectively address the challenges faced by women in similar contexts. These contexts include individuals with low income, social vulnerability, affiliation with health insurance entities catering to those without payment capacity, unemployment, and countries with fragmented healthcare systems [24].

We believe that analyzing barriers based on the social determinants of health, while integrating multiple dimensions of adherence, proves beneficial for devising intervention strategies. Our study examined social and economic factors, the health conditions of women, and factors associated with both adherent and non-adherent individuals. Among the women who participated in the program, we identified barriers related to the healthcare team, the healthcare system itself, and factors pertaining to the patients. It is important to note that these classifications did not generate completely distinct or mutually exclusive categories. Instead, they provided a framework for understanding phenomena that could operate at various levels simultaneously. Addressing these issues at the personal level necessitates actions targeting the structural or intermediate determinants.

Fragmented healthcare networks operate through intricate administrative mechanisms, which impede an effective response in terms of cancer prevention [10, 25]. This study was conducted in a low-income context and successfully identified these barriers, underscoring the necessity to implement system-level improvements to achieve greater integration of health services. Similarly, a previous study reported the existence of similar barriers among women residing in areas with varying degrees of deprivation [26]. Through the accounts of women and testimonials from healthcare personnel, our study provides a deeper understanding of how women living in low-income environments respond to the challenges of daily life. We believe that the difficulties they encounter result in distinct responses compared to their counterparts from higher socioeconomic strata. The presence of greater hardships or fewer advantages to benefit from preventive programs constitutes a disparity of significant importance when designing strategies to enhance attendance. The absence of robust policies and programs aimed at safeguarding the well-being of impoverished women can have even more profound consequences for their health, given the cumulative impact of risks over the course of a lifetime.

One barrier to cancer diagnosis observed in low- and middle-income countries is disease literacy, which is linked to a low level of education [27]. In our study, what we refer to as living conditions may align with what other researchers have identified as the prioritization of competing demands [11, 28, 29]. Our understanding is grounded in recognizing the characteristics of women with a low socioeconomic status who possess limited knowledge about cervical cancer prevention and lack a support network within their families to assist with caregiving responsibilities. This group of women often face obstacles in making autonomous decisions regarding undergoing cervical cancer screening tests. However, this situation is not confined to isolated cases; rather, it is associated with a multifaceted context. In the absence of community resources or social programs targeting women, this issue recurs frequently throughout their lives, showcasing cumulative and detrimental effects of SDH. Therefore, we provide a more precise definition of this category below.

Life conditions. Although many specific phenomena affect adherence, the discourse of the female participants reveals a common axis associated with the SDH, which deteriorates the individual’s capacity to decide on their self-care. The influence of structural SDH operates as a complex interwoven system composed of social protection policies based on minimal support networks, precarious work, the weakness of education policies that result in a lack of work opportunities, and high rates of unemployment, among others [30, 31]. The effects of structural SDH in low-income contexts are expressed to such an extent that providing health services to vulnerable populations requires reflection and a better understanding of the phenomenon of adherence in social and population-level contexts. This will allow the generation of effective collective strategies that can be implemented within the structure of welfare programs, rather than isolated and unsustainable interventions within the context of healthcare networks.

Distance to health services: The findings from our study lead us to conclude that the proximity of healthcare facilities for cytology tests plays a critical role in the decision-making process regarding attendance at screening. Women residing in remote areas identified distance as a substantial barrier to their adherence to screening. The presence of medical missions in the region emerged as a vital enabler to reach non-adherent women and enhance their participation in the screening program [32]. Distance and women’s relationship with health services are factors that interact with each other, which allows us to understand that the decisions to attend become complex in association with many circumstances.

Relationship with health services. The narratives of women in our study emphasized the significance of utilizing primary care services in general, specifically in relation to attending cervical cytology screenings. Our findings align with existing literature that highlights regular utilization of medical services as a facilitator for program accessibility [33],, particularly when it is associated with routine procedures. Additionally, women expressed positive valuations towards having a female examiner, receiving exclusive attention from a doctor dedicated to the program, and receiving quality care. These attributes played a crucial role in promoting adherence. Our results are consistent with other studies conducted in Latin American low-income settings, which have identified similar barriers to the implementation of cervical cancer prevention technologies [10, 34].

Perceptions on the preventive program for cervical cancer: An important area for improvement in Colombia is to achieve a 70% screening coverage, utilizing a high-precision test, among women affiliated with subsidized health insurance before the age of 35 and again before the age of 45. Additionally, it is necessary to develop efficient mechanisms to assess a woman’s compliance with screening rounds promptly [35]. Furthermore, the implementation of an information system is required to track women who do not attend the screenings. Women demand the need to develop patient-centered care [36]. For this reason, the research team will continue to advance in the design of interventions in response to this need in the future [37].

The research was carried out in the context of opportunistic screening, where individual initiative or doctor’s recommendation to attend screening is very important. As we mentioned before, a characteristic of the living conditions of women beneficiaries of the program is the low educational level. To achieve better results in screening coverage, structural changes in health policy must be considered; the transformation towards organized preventive programs, which offer novel strategies to invite women to participate in screening rounds, among other activities, are effective strategies to influence women’s education, educational inequalities are significantly lower in countries with population-based detection [38].

Our findings reveal that traditional values and beliefs rooted in the culture of indigenous women act as a significant obstacle to their participation in the cervical cancer prevention program [39]. To address this, we propose exploring self-testing interventions that are more acceptable to these communities [40]. In addition, future research should analyze the role of religion in shaping these attitudes, as fatalistic ideas may present significant barriers to access [41]. Fear and shame emerged as significant factors that deter women from participating in the program, which is a common barrier experienced by women worldwide [30, 42–46]. Many women expressed apprehension about the procedure itself, with stories from other women and past experiences, particularly those related to the use of the speculum, exacerbating their fears. Additionally, they expressed a feeling of shame, especially if the person taking the sample is a man [42].

Our findings are consistent with previous research that has identified barriers to screening in low-income settings. These barriers operate at different levels, including the individual, social, and cultural/religious domains [47]. The adherence to the cytology test is influenced by gender roles, as partners’ support or prohibition of attending screening plays an important role in women’s decision to participate in preventive programs [42]. Moreover, self-care associated with the reproductive role becomes a barrier for single women or those who have completed their reproductive cycle, as they perceive a lower risk. Cultural factors did not show a significant influence on promoting attendance for the cytology test in our findings. However, the impact of culture is complex and highlights the need for health services staff to strengthen their training in intercultural competencies, allowing them to detect cultural aspects and provide differential treatment to each woman.

The healthcare personnel have identified the existing barriers in the RSL and have implemented several improvement mechanisms in recent years. These mechanisms include monitoring cases of women with abnormalities and providing care through campaigns in remote areas. However, it is also crucial to identify non-adherent women who seek healthcare services from other facilities within the network. To accomplish this, enhancements in information systems and the adoption of new technologies are necessary to enhance women’s engagement with primary care services and promote their commitment to their own health. Additionally, strategies are needed to encourage women who have never attended the network to undergo cytology screening, taking into consideration their social and cultural characteristics that may influence their attendance. Furthermore, more population-based studies are required to explore factors that enhance the acceptability of HPV-based screening [48].

Diseases that primarily affect impoverished populations can be marginalized in countries’ agendas, as is the case with cervical cancer [49], posing a risk of losing investments in new technologies such as HPV vaccination, despite evidence of its population-level impact [50]. To enhance cytology coverage in underserved populations, a combination of intersectoral actions addressing population education and sector-specific measures regarding program organization is required. In populations with lower educational levels, preventive programs need to exert greater efforts to ensure women comprehend cancer prevention mechanisms and can make informed decisions regarding screening attendance. Moreover, sector-specific actions are needed to establish evidence-based, organized population screening programs [38].

Cervical cancer screening programs and sociocultural norms vary among populations, giving rise to different reasons or barriers that contribute to non-attendance or low adherence to the program. Therefore, it is essential to conduct specific studies in each country [50, 51] to identify the unique factors associated with population clusters and their diverse needs. This study provides evidence on the obstacles faced by women when accessing cytology screening and highlights common aspects observed in populations with low screening rates. These aspects include limited educational opportunities, resource constraints, absence of support networks, fragmented healthcare systems, and opportunistic screening programs. Additionally, personal reasons such as fear, shame, and discomfort are universally encountered by women worldwide. Moreover, cultural factors such as ethnicity, religion, and gender roles play a role and necessitate the development of intercultural care models. Understanding women’s interactions with primary care services and the impact of distance to healthcare facilities are crucial aspects that require further investigation to enhance our understanding of their influence on cytology adherence.

Our results indicate that gender roles have an influence on adherence to cytology screening, acting as a facilitator for access among women who assume a reproductive role and have a sexual partner. Conversely, it acts as a barrier for single women or those who have completed their reproductive cycle, as they exhibit a low perception of the risk of developing cancer. The impact of various factors such as advertising campaigns and personal influences from family members and close acquaintances facilitates adherence. Our findings support this evidence, although we did not observe a significant influence of cultural factors in promoting attendance for cytology screening.

The reasons for non-attendance in our study were consistent with those reported in the literature for underserved populations. Barriers to adherence were identified across various dimensions, including social and economic factors, health conditions, and patient-related factors, affecting both adherent and non-adherent women. Among women who had attended the program, we identified barriers and facilitators related to the healthcare team and system, as well as patient-related factors.

The healthcare personnel have identified the barriers that exist in the RSL and hinder the attainment of improved outcomes in the cervical cancer screening program at the population level. To mitigate the risk of cervical cancer, it is necessary to implement strategies such as monitoring cases with abnormalities and providing screening services to non-adherent women in targeted groups. Accomplishing this goal will necessitate enhancing information systems and incorporating new technologies that facilitate better engagement with women [46]. By addressing the challenges associated with the barriers to adherence to cervical cancer prevention program in the RSL, we can enhance screening outcomes for women in the population and thus, contribute to the national and global goal of screening 70% of the target population of the program within the global strategy to accelerate the elimination of cervical cancer defined by the World Health Organization by [52].

## Conclusions

The findings of this study provide valuable insights into the functioning of cervical cancer prevention programs in local and low-income settings. The qualitative case study sheds light on the program’s operations within the framework of care networks, where the responsibility of managing population risks at both individual and collective levels is shared between the care network and the health insurance entities. Based on our findings, it is evident that there is a need to enhance the efforts of health insurance entities in establishing effective communication channels with women participating in the cervical cancer prevention program. One notable barrier to proper case follow-up arises from the lack of information systems and adequate communication processes that facilitate contact between women enrolled in health insurance and healthcare network officials.

Healthcare personnel have highlighted the existing barriers in the RSL that hinder achieving better outcomes in the population-level cervical cancer screening program. It is necessary to strengthen case follow-up actions for cases with abnormalities and implement strategies to offer screening services to groups of non-adherent women. To achieve this, it would be beneficial to strengthen information systems and adapt new technologies that enable more effective communication with the population, in order to motivate women to actively engage in their own healthcare. Overcoming the identified barriers requires increasing the capacity of healthcare personnel to provide intercultural interventions. Additionally, incorporating innovative processes that support women in overcoming fear, shame, and lack of knowledge about the cytology procedure is crucial to promote greater adherence to cytology in the RSL. Furthermore, urging local and national authorities to implement intersectoral actions that promote women’s education, population-based screening, and modernization of information systems to manage the program are highly necessary improvements to achieve a greater impact on the program’s beneficiary population.

### Public health implications

Cervical cancer can be reduced through the integration of population-based screening and HPV virus vaccination strategies [53], provided that high coverage rates are achieved for both interventions. The decline in acceptance of cytology screening is a pressing issue in countries with well-established preventive programs, while even more challenging is the lack of knowledge in countries with fragmented health systems that have yet to implement widespread vaccination efforts. Given these circumstances, it is crucial to conduct an analysis of barriers to generate knowledge that, when coupled with targeted actions, can lead to higher levels of cervical cancer prevention.

Considering adherence as a social phenomenon within the framework of SDH can broaden our understanding of adequate preventive measures in low-income contexts. This cumulative phenomenon affects not only women’s adherence to cytology, but also health in general, as could be identified in the accounts of the participating women. Therefore, it is imperative to advocate for women’s right to comprehensive health and recognize the social context in which adherence occurs. This approach may allow the development of effective population-level interventions that can be integrated into existing health care programs, or even include training strategies in school education programs. These interventions aim to improve general awareness of the value of cancer screening [54] and promote intersectoral actions that enhance individuals’ and communities’ agency capacity. The evidence generated regarding the barriers faced by low-income women is a valuable resource for developing effective and contextually relevant interventions.

### Limitations

Our study specifically targeted women who were beneficiaries of health insurance and fell into the category of being poor and unemployed. Regrettably, we were unable to incorporate women without health insurance into our research due to the unavailability of medical records containing their screening history and past engagements with primary healthcare services. Consequently, our study did not encompass a high-risk population that could have provided significant insights and recommendations for enhancing health policies. This exclusion represents a constraint in our study, and future research endeavors should aim to address this gap.

### Strengths

Although numerous studies have investigated barriers to cytology screening in high-income countries [36], the majority of them have primarily focused on underserved populations [55, 56]. However, there is a scarcity of comprehensive studies examining adherence in low-income contexts. This research aims to address this gap by presenting a multidimensional analysis of cytology adherence screening in the context of SDH. By considering various factors that influence adherence, this study offers a deeper understanding of the mechanisms that impact cytology adherence among women who benefit from subsidized health insurance. The findings from this research can inform the development of tailored interventions and strategies to enhance adherence and improve screening outcomes in low-income settings.

While numerous studies have investigated barriers to cytology screening in high-income countries [51, 57, 58], most of them have focused on underserved populations [28]. There are, however, very few studies that provide a comprehensive understanding of adherence in a low-income context. The research contributes to bridging this gap by presenting a multidimensional analysis of cytology adherence screening in relation to SDH. By considering various factors that influence adherence, this study provides a deeper understanding of the mechanisms that affect cytology adherence in a low-income context. The findings of this research can be useful in developing tailored interventions and strategies to improve adherence and screening outcomes in low-income settings.

## Data Availability

The datasets generated and/or analysed during the current study are not publicly available due a condition of the endorsement of the Ethics Committee for Research, but are available from the corresponding author on reasonable request.

## List of abbreviations

HPV: High-risk papillomavirus
SDH: Social Determinants of Health
RSL: Ladera Health Network
FG: Focus group discussions
DI: Depth interviews
SI: Semi-structured interview
